# Urinary TWEAK reflects disease activity in ANCA-associated vasculitis

**DOI:** 10.1093/ckj/sfaf086

**Published:** 2025-04-18

**Authors:** Ásta Dögg Jónasdóttir, Peter Hemmingsson, Angelina Schwarz, Magnus Söderberg, Annika Wernerson, Abdul Rashid Qureshi, Aleksandra Antovic, Iva Gunnarsson, Annette Bruchfeld

**Affiliations:** Department of Clinical Science, Intervention and Technology, Division of Renal Medicine, Karolinska Institutet, Stockholm, Sweden; Division of Nephrology, Department of Medicine, Landspitali - The National University Hospital, Reykjavik, Iceland; Department of Clinical Science, Intervention and Technology, Division of Renal Medicine, Karolinska Institutet, Stockholm, Sweden; Department of Clinical Science, Intervention and Technology, Division of Renal Medicine, Karolinska Institutet, Stockholm, Sweden; Pathology, Clinical Pharmacology and Safety Sciences, BioPharmaceuticals R&D, AstraZeneca, Gothenburg, Sweden; Department of Clinical Science, Intervention and Technology, Division of Renal Medicine, Karolinska Institutet, Stockholm, Sweden; Department of Clinical Science, Intervention and Technology, Baxter Novum, Karolinska Institutet, Stockholm, Sweden; Department of Medicine, Division of Rheumatology Solna, Karolinska Institutet, Stockholm, Sweden; Unit of Rheumatology, Karolinska University Hospital, Stockholm, Sweden; Department of Medicine, Division of Rheumatology Solna, Karolinska Institutet, Stockholm, Sweden; Unit of Rheumatology, Karolinska University Hospital, Stockholm, Sweden; Department of Clinical Science, Intervention and Technology, Division of Renal Medicine, Karolinska Institutet, Stockholm, Sweden; Department of Health, Medicine and Caring Sciences, Linköping University, Linköping, Sweden

**Keywords:** ANCA-associated vasculitis, biomarker, BVAS, urinary TWEAK

## Abstract

**Background:**

The aim of the study was to investigate urinary and serum tumour necrosis factor (TNF)-like weak inducer of apoptosis (TWEAK) as potential biomarkers in a longitudinal cohort of patients with ANCA-associated vasculitis (AAV).

**Methods:**

Patients with active AAV were included in the study. The Birmingham Vasculitis Score 2003 (BVAS) was used for assessment of disease activity and C-reactive protein (CRP), creatinine, albuminuria, and serum (s) and urinary (u) TWEAK levels were measured at baseline and 6-month follow-up. sTWEAK was measured in population-based controls for comparison. Kidney biopsies from AAV patients were stained for TWEAK and its receptor fibroblast growth factor-inducible 14 (Fn14) using immunohistochemistry (IHC).

**Results:**

sTWEAK was measured in 74 patients and uTWEAK in 69 patients, 42 of whom had kidney involvement. uTWEAK-to-creatinine ratio (uTWEAK/Cr) was significantly higher at baseline compared with follow-up (median 7.21 vs 4.94 ng/mmol, *P* < .0001). Patients with kidney involvement had higher uTWEAK/Cr levels compared with those without (*P* = .03). A correlation was found between uTWEAK/Cr and BVAS (*P* = .006), albuminuria (*P* = .022) and crescentic changes (*P* = .03). sTWEAK levels were higher in patients at inclusion than at follow-up (*P* = .009) but no difference was found when comparing patients and controls, nor did sTWEAK correlate with BVAS. IHC staining showed a clear expression of TWEAK but a fainter pattern of Fn14 in kidney biopsies from AAV patients.

**Conclusions:**

uTWEAK/Cr correlated with BVAS, albuminuria and number of crescents in active AAV and may be a useful biomarker in assessing disease activity in patients with AAV, whereas sTWEAK level is not.

## Graphical Abstract

**Graphical Abstract:**
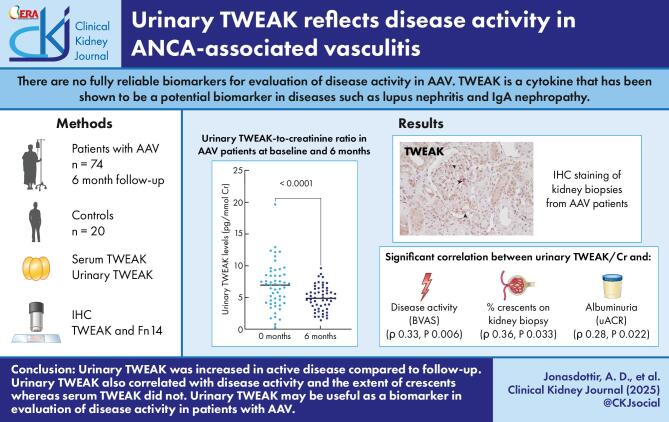

KEY LEARNING POINTS **What was known:**

- There are as of yet no fully reliable biomarkers for evaluation of disease activity in AAV. TWEAK is a cytokine that has been shown to be a potential biomarker in diseases such as lupus nephritis and IgA nephropathy but has not previously been studied in AAV.

**This study adds:**

- Urinary TWEAK levels in AAV patients followed longitudinally were significantly higher in active disease compared with remission and correlated with BVAS and the extent of crescents on kidney biopsies. A correlation was found between urinary TWEAK and albuminuria, and urinary TWEAK levels were higher in patients with kidney involvement. Immunohistochemical staining showed expression of TWEAK and a fainter pattern of Fn14 in kidney biopsies from AAV patients.

**Potential impact:**

- Urinary TWEAK may thus be useful as a biomarker in evaluation of disease activity in patients with AAV.

## INTRODUCTION

Anti-neutrophil cytoplasmic antibody (ANCA)-associated vasculitis (AAV) is a group of necrotizing vasculitides characterized by inflammation of small- to medium-sized vessels and the presence of ANCAs [1]. The clinical presentation varies greatly, ranging from localized manifestations to multisystem organ involvement. Kidney involvement is common in AAV and can lead to kidney failure with the need for kidney replacement therapy [2, 3]. Disease flares are frequent, with >50% of patients experiencing a relapse within 5 years from diagnosis [2, 4, 5]. Simple and non-invasive methods for assessment of kidney disease activity are lacking, rendering follow-up of these patients challenging.

Tumour necrosis factor (TNF)-like weak inducer of apoptosis (TWEAK) is a type II transmembrane glycoprotein that belongs to the TNF cytokine superfamily. TWEAK is cleaved by proteolysis and circulates in the plasma in a soluble form in trimers. TWEAK expression has been demonstrated in various inflammatory cells such as dendritic cells, monocytes, and natural killer cells [6–8] as well as in tubular and endothelial cells in the kidney [9, 10]. TWEAK binds to its receptor, fibroblast growth factor-inducible-14 (Fn14) [11]. Fn14 is expressed by various cells, including endothelial and epithelial cells [12] as well as podocytes and tubular and mesangial cells in the kidney [13]. The expression of Fn14 is low in healthy tissues but is upregulated in tissue injury [14]. The effects of TWEAK/Fn14 vary depending on circumstances and cells involved but include upregulation of inflammatory cytokines [15], induction of endothelial cell survival and proliferation [16], cell death by apoptosis and necrosis [17, 18], and induction of fibrogenesis [19].

Increased expression of TWEAK and its receptor Fn14 has been described in the settings of acute and chronic kidney injury [20]. Fn14 expression is upregulated after kidney injury, and TWEAK exerts enhanced pro-inflammatory effects in these settings [13, 21].

Elevated serum TWEAK (sTWEAK) levels have been shown to correlate with disease activity in systemic lupus erythematosus (SLE) [22, 23]. Furthermore, urinary TWEAK (uTWEAK) has been suggested to be a potential biomarker in lupus nephritis (LN) and IgA nephropathy (IgAN) [24, 25].

The aim of this study was to investigate the role of serum and urinary TWEAK as potential biomarkers of disease activity and markers of kidney involvement in a longitudinal cohort of patients with AAV.

## MATERIALS AND METHODS

### Patients and methods

Patients with active AAV from the Departments of Nephrology and Rheumatology at Karolinska University Hospital in Stockholm were included close to the start of induction treatment for either newly diagnosed AAV, or a disease flare. Patients were followed prospectively for 6 months. Serum samples from a population-based cohort were used as a disease-free control group [26].

### Disease activity and kidney involvement

The Birmingham Vasculitis Score 2003 (BVAS) [27] was used for assessment of the disease activity. Disease remission was defined as a BVAS of 0. Kidney biopsy consistent with pauci-immune-vasculitis or clinical findings of affected kidney function and/or significant haematuria (≥2+ on dipstick urinalysis and/or >10 erythrocytes per high-power field on urinary sediment) was defined as kidney involvement.

### Treatment

Remission induction and remission maintenance therapy were documented. The cumulative glucocorticoid (GC) doses given before baseline sampling were calculated in prednisolone-equivalent milligrams and daily doses at inclusion and follow-up were recorded. In patients included at the time of a disease relapse, low-dose GCs (up to 10 mg) given before the relapse as maintenance therapy were not included in the calculation.

### Ethics

The study was conducted in accordance with the Declaration of Helsinki and the study protocol was approved by the Regional Ethical Review Board in Stockholm and the Swedish Ethical Review Authority. A written informed consent was obtained from all participants at inclusion.

### Laboratory analysis

Peripheral venous blood and urine samples were frozen and stored at −70°C for future analysis. Routine laboratory analyses were carried out using standard methods at the Karolinska University Hospital, including serum C-reactive protein (CRP) plasma creatinine, urine albumin-to-creatinine ratio (mg/mmol), and urinalysis. Urine kappa, lambda, and protein HC (α1-microglobulin) were measured using standard methods at the Karolinska University Hospital and normalized to urine creatinine concentrations. Estimated glomerular filtration rate (eGFR) was calculated using the Chronic Kidney Disease Epidemiology Collaboration (CKD–EPI) equation [28].

Analysis of ANCA serology was performed with an enzyme-linked immunosorbent assay (ELISA) (direct ELIA, Euro Diagnostic) or a multiplex (BioPlex™ 2200, Bio-Rad) according to routine clinical practice at the Department of Clinical Immunology at Karolinska University Hospital. sTWEAK and uTWEAK levels were measured with a commercially available ELISA kit from Bioscience (Hatfield, UK). uTWEAK levels were normalized to urine creatinine concentrations measured in the same urine sample (ng/mmol).

### Immunohistochemical staining

Kidney biopsies from two AAV patients were stained using immunohistochemistry (IHC). Macroscopically unaffected tissue from nephrectomized kidney cancer patients was used as a control. The biopsies and tissues were paraffin-embedded and sectioned into 1.5-µm thick sections at the Pathology Kidney Laboratory at Karolinska University Hospital. For IHC the avidin/biotin blocking kit (Vector Laboratories SP2001) and DAB Substrate Kit, Peroxidase (Vector Laboratories SK-4100) were used according to the manufacturer's guidelines. Previous deparaffinization and hydration were performed through washing steps of xylene and ethanol (100%, 95%, 70%) and MilliQ. Antigen retrieval was achieved by microwaving the slides for 25 min in 10 mM citrate buffer (pH 6). Primary antibodies anti-human TWEAK R/Fn14 (R&D Systems, AF1199) and anti-TWEAK (Bioworld Technology, Inc., BS2454) were used at 4°C overnight, and as secondary antibodies horse anti-goat IgG antibody (H + L), biotinylated (Vector Laboratories, BA-9500-1.5), and goat anti-rabbit IgG, biotinylated (Vector Laboratories, BA-1000-1.5), were used.

### Histopathological classification score

A histopathological classification score (Berden score) for AAV that distinguishes the pattern of kidney injury in four classes – focal, mixed, sclerotic, and crescentic – was used to score all available kidney biopsies [29]. An experienced kidney pathologist (M.S.), blinded to patient data, scored the biopsies. Kidney biopsy reports were also reviewed for percentage of crescents.

### Statistics

Normally distributed variables are presented as mean and standard deviation (SD), non-normally distributed variables as median and 25th–75th percentiles, and categorical variables as frequency and percentage. The non-parametric Wilcoxon signed rank test was used for comparison between time points, the non-parametric Wilcoxon rank sum test to assess the difference between groups, and Fisher's exact test for comparison of categorical data. Correlations were analysed using the non-parametric Spearman rank test. Statistical significance was defined as *P* < .05. Statistical analysis was performed with JMP software (version 14, SA Campus Drive, Cary, NC, USA) and GraphPad Prism (version 9, GraphPad Software, La Jolla, CA, USA).

## RESULTS

### Patients

Seventy-four patients with active AAV were included in the study (Table 1). All patients were ANCA-positive, 47 for proteinase-3 (PR3) ANCA and 27 for myeloperoxidase (MPO) ANCA. For comparison, sTWEAK levels were also measured in 20 population-based controls. The baseline characteristics of patients and controls and demographic data are shown in Table 1.

**Table 1:** Patient and control characteristics at baseline.

**Variable**	**AAV patients** ***n* = 74**	**Controls** ***n* = 20**	***P***
Age, years	58 (48–69)	64 (59–70)	.12
Sex, female/male	32 (43.2)/42 (56.8)	6 (30)/14 (70)	.32
ANCA			
PR3	47 (63.5)		
MPO	27 (36.5)		
Diagnosis			
GPA	53 (71.6)		
MPA	17 (23.0)		
EGPA	4 (5.4)		
New diagnosis	65 (87.8)		
Relapse	9 (12.2)		
Kidney involvement	42 (56.7)		
Treatment prior to inclusion			
GCs	67 (90.6)		
Intravenous GCs	29 (39.2)		
Median cumulative GC dose at baseline, mg	525 (150–1875)		
Cyclophosphamide	21 (28.4)		
Rituximab	3 (4.1)		
Methotrexate	6 (8.1)		
Mycophenolate mofetil	2 (2.7)		
Induction treatment			
Cyclophosphamide	47 (63.5)		
Rituximab	16 (21.6)		
Methotrexate	12 (16.2)		
Mycophenolate mofetil	5 (6.8)		

Data are shown as median (25th–75th percentile) or number (percentage)

ANCA, anti-neutrophil cytoplasmic antibody; PR3, proteinase-3; MPO, myeloperoxidase; GPA, granulomatosis with polyangiitis; MPA, microscopic polyangiitis; EGPA, eosinophilic granulomatosis with polyangiitis; GCs, glucocorticoids.

### Treatment

At inclusion all except seven patients had commenced induction treatment at the time of sampling (median 5 days, range 0–37 days). Treatment is shown in Table 1. At the 6-month follow-up, remission maintenance treatment with azathioprine, mycophenolate mofetil, methotrexate or rituximab had been initiated in 55 (74.3%) of the patients.

### Disease activity and phenotype

The median BVAS at baseline was 15 and decreased to a median of 0 at the 6-month follow-up (*P *< .0001). Most of the patients had multi-organ involvement at inclusion but six patients had only localized ear, nose and throat manifestations. Forty-two (56.8%) of the patients had kidney involvement at baseline, in 39 patients confirmed with a kidney biopsy and in the remaining three by fulfilling the criteria for a clinical diagnosis (Table 1). At follow-up, all except five patients were in remission (BVAS 0); none had active kidney involvement.

### Laboratory results

#### Urinary TWEAK

**Figure 1:**
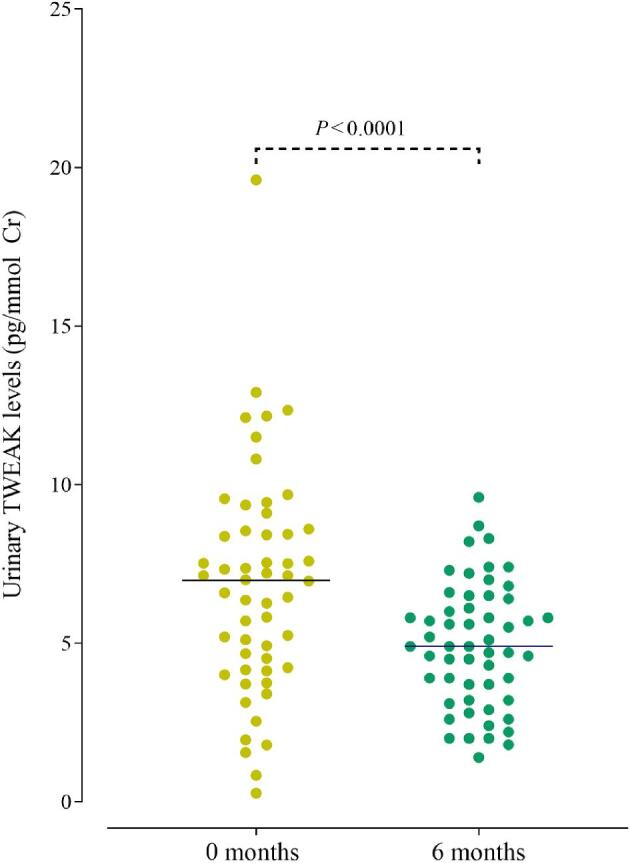
Urinary TWEAK-to-creatinine ratio in ANCA-associated vasculitis patients at baseline (0 months) and 6-month follow-up. Non-parametric Wilcoxon signed rank test.

Urine samples for measurement of TWEAK-to-creatinine ratio (uTWEAK/Cr) were available in 69 of the subjects. uTWEAK/Cr levels were higher at baseline compared with follow-up (median 7.21 vs 4.94 ng/mmol, *P* < .001) (Table 2 and Fig. 1). uTWEAK/Cr was higher at baseline in patients with kidney involvement compared with those without (median 8.49 vs 6.34 ng/mmol, *P* = .03). PR3-ANCA-positive patients showed similar uTWEAK levels compared with MPO-ANCA-positive patients. There was no significant difference in uTWEAK/Cr between patients in remission and the five patients who remained active at 6 months.

**Table 2:** BVAS and laboratory variables at baseline and at the 6-month follow-up.

**Variable**	**Baseline**	**6 months**	***P***
BVAS	15 (9.8–21.0)	0 (0.0–0.0)	**<.0001**
Plasma creatinine, μmol/L	86 (69.0–144.3)	91.5 (80–124.5)	.93
eGFR, mL/min/1.73 m^2^^a^	79.5 (37.7–102.8)	71.3 (45.8–89.3)	.06
Serum CRP, mg/L^b^	10 (3.0–28.0)	2 (0–4.3)	**<.0001**
Haematuria, no. (%)^c^	42 (56.8)	11 (15.3)	**<.0001**
UACR, mg/mmol	8.95 (0.9–25.8)	3.0 (0.7–15.0)	**<.0001**
Serum TWEAK, pg/mL	465.6 (356.9–707.3)	436.1 (347.6–547.3)	**.009**
Urinary TWEAK, ng/mmol Cr^d^	7.2 (4.6–9.6)	4.9 (3.6–6.5)	**<.0001**

Data are shown as median (25th–75th percentiles) except for haematuria, which is shown as number (percentage). Wilcoxon signed rank test was used except for haematuria, for which Fisher´s exact test was used. Statistically significant values (*P* < .05) are shown in bold type.

BVAS, birmingham vasculitis score 2003; eGFR, estimated glomerular filtration rate; CRP, C-reactive protein; UACR, urinary albumin-to-creatinine ratio; TWEAK, tumour necrosis factor (TNF)-like weak inducer of apoptosis.

a Calculated using the CKD–EPI equation.

b CRP normal reference range <3 mg/L.

c Haematuria was defined as 2+ on urine dipstick or >10 erythrocytes/HPF on urine sediment. Urinanalysis and/or urine sediment was available in 74 patients at baseline and 72 patients at follow-up.

d Urinary TWEAK ng/mmol Cr ratio was available in 69 patients at baseline and at follow-up.

**Figure 2:**
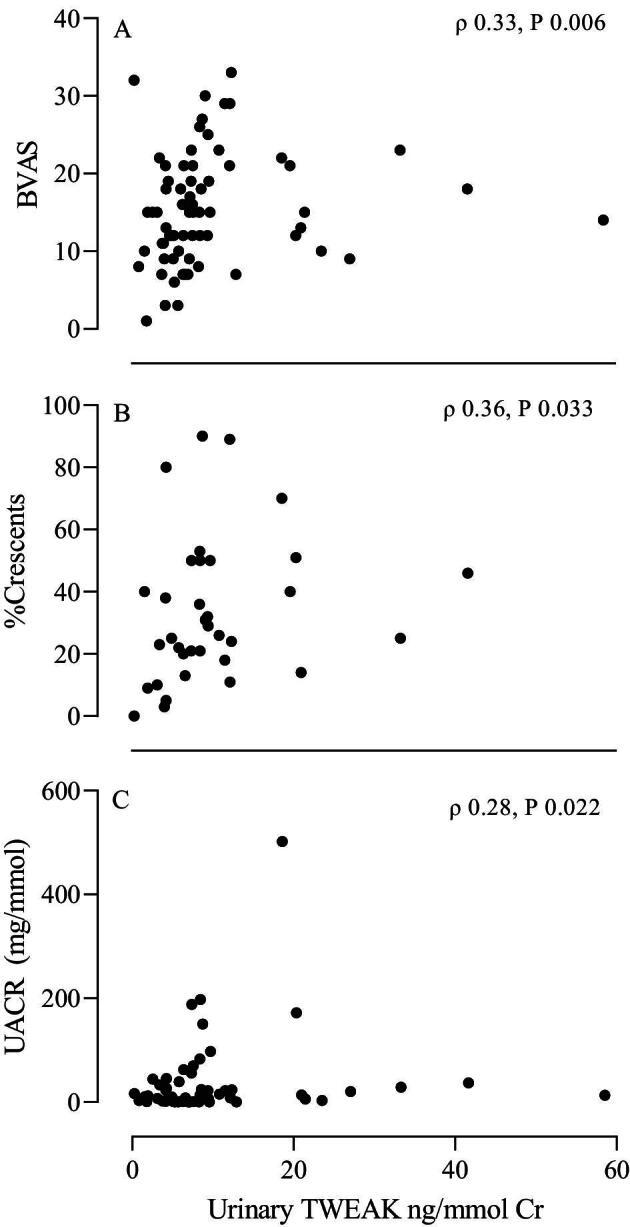
Correlation between urinary TWEAK-to-creatinine ratio and (A) disease activity (BVAS), (B) percentage crescents on kidney biopsy, and (C) urinary albumin-to-creatinine ratio (UACR) at baseline. Spearman correlation coefficient.

A correlation was found between uTWEAK/Cr and BVAS (*ρ* = 0.33, *P* = .006) and between the grade of albuminuria and uTWEAK/Cr (*ρ* = 0.28, *P* = .022) at baseline (Table 3 and Fig. 2). No difference in uTWEAK/Cr levels between patients with and without significant haematuria at baseline or at follow-up was found. No correlation was seen between uTWEAK/Cr and sTWEAK levels. Measurement of the low molecular weight proteinuria markers urine kappa, lambda, and protein HC was available in nine patients with kidney involvement at baseline. No correlation was found between any of these urine proteins and TWEAK/Cr (data not shown).

**Table 3:** Spearman rank correlation coefficient for serum/urinary TWEAK and laboratory and clinical variables at baseline in patients with AAV.

	**Serum TWEAK (pg/mL**)		**Urinary TWEAK/Cr (ng/mmol)**	
**Variable**	***ρ***	***P***	***ρ***	***P***
BVAS	−0.062	.596	0.327	**.006**
Serum CRP (mg/L)	0.009	.942	0.079	.519
Plasma creatinine (μmol/L)	−0.326	**.005**	0.180	.138
eGFR^a^ (mL/min/1.73 m^2^)	0.313	**.007**	−0.208	.087
UACR (mg/mmol)	−0.146	.234	0.277	**.022**
Serum TWEAK (pg/mL)			−0.156	.201
Urinary TWEAK ng/mmol Cr	−0.156	.201		
Cumulative GC dose^b^ (mg)	−0.267	**.021**	0.281	**.019**
Percentage crescents	−0.288	.07	0.362	**.033**

Statistically significant values (*P* < .05) are shown in bold type.

BVAS, birmingham vasculitis score 2003; CRP, C-reactive protein; eGFR, estimated glomerular filtration rate; UACR, urinary albumin-to-creatinine ratio; TWEAK, tumour necrosis factor (TNF)-like weak inducer of apoptosis; GC, glucocorticoids.

a Calculated using the CKD–EPI equation.

b Prednisolone equivalent milligrams.

#### Serum TWEAK

sTWEAK levels were significantly higher at baseline compared with follow-up (median 465.6 vs 436.1 pg/mL, *P* = .009) (Table 2). However, no significant differences were found between sTWEAK levels in controls compared with patients at baseline (*P* = .67) or follow-up (*P* = .054). Moreover, no differences were found in sTWEAK levels in remission and in those with active disease at follow-up or in patients with kidney involvement compared with those without.

No association was noted between sTWEAK levels and BVAS at inclusion but a correlation was found between sTWEAK levels and creatinine (*ρ* = −0.326, *P* = .005) and eGFR (*ρ* = 0.313, *P* = .007) (Table 3)

#### CRP levels

CRP decreased significantly from inclusion to follow-up (median 10 vs 2 mg/L, *P* < .0001) (Table 2). There was neither a correlation between CRP and BVAS at baseline nor one between CRP and sTWEAK levels or uTWEAK/Cr (Table 3).

#### Treatment and TWEAK levels

The uTWEAK/Cr levels at baseline were significantly higher in patients who had received immunosuppressive treatment at inclusion compared with those who were treatment-naive (7.5 vs 5.7 ng/mmol, *P* = .03). No difference was found in sTWEAK levels between these groups.

A significant correlation was found between the cumulative GC dose at inclusion and uTWEAK/Cr (*ρ* = 0.28, *P* = .02) and a negative correlation between the cumulative GC dose at inclusion and sTWEAK levels (*ρ* = −0.27, *P* = .02) (Table 3). No association was observed between sTWEAK or uTWEAK/Cr and the daily GC dose at the 6-month follow-up.

There was no difference in sTWEAK levels between patients who had received treatment with cyclophosphamide at inclusion compared with those who had not. However, uTWEAK/Cr levels were higher in patients who had received cyclophosphamide compared with those who had not (9.35 vs 6.30 pg/mL, *P* = .005). No differences were observed between other treatments groups.

#### Immunohistochemical staining

**Figure 3:**
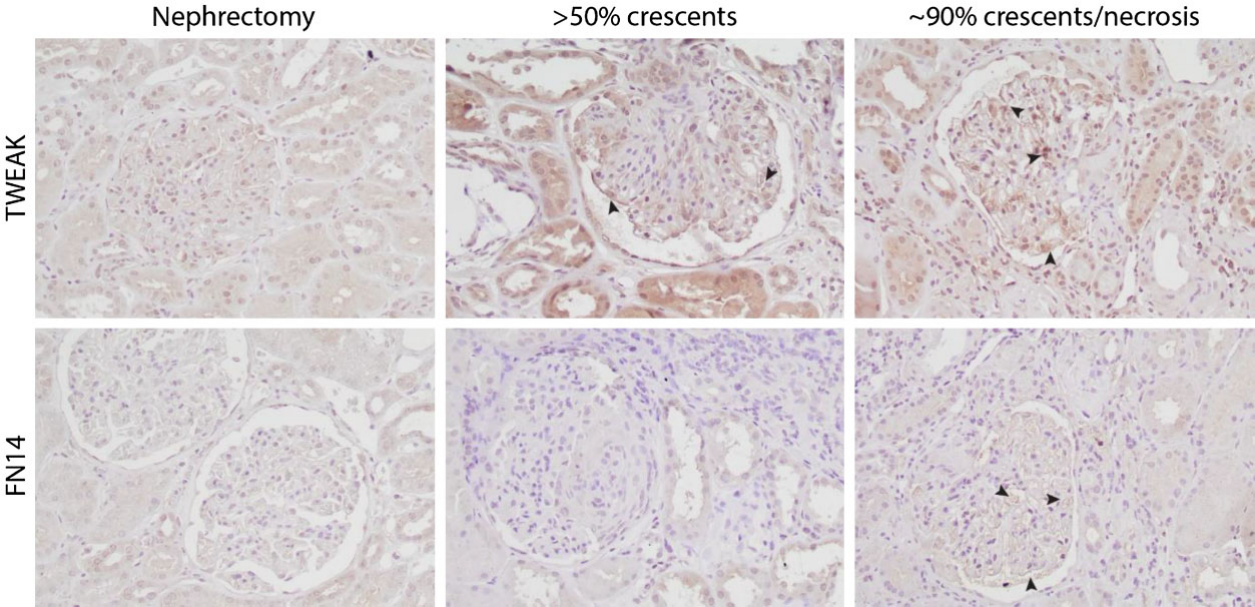
Immunohistochemical staining of kidney biopsies from patients with ANCA-associated vasculitis (AAV) against TWEAK and its receptor, Fn14. Both anti-TWEAK and anti-Fn14 show increased stain for the patients’ biopsies with crescentic manifestations of AAV compared with the control nephrectomy tissue. The arrows highlight a few points of expression in the glomeruli and podocytes. Magnification 20×.

Kidney biopsies from two active AAV patients were stained using IHC, one with ∼90% crescents/necrosis and one with >50% crescents. The staining showed a clear expression of TWEAK in the kidney biopsies from AAV patients, involving both glomerular and tubular areas when compared with control kidney tissue. In the glomeruli we found TWEAK accumulation in the formed crescents and podocyte cell body as well as a distinct linear staining pattern, indicating expression in the podocyte foot processes. There was TWEAK expression in circular shapes of endothelial cells lining the capillary walls. Both proximal and distal tubules exhibited increased TWEAK expression. Evaluating TWEAK's receptor, Fn14, we found a slight increase in expression mainly in the kidney sample from the AAV patient with severe crescent/necrosis (around 90%). The control tissue showed faint podocyte and endothelial glomerular staining but in the severe AAV case there was upregulated expression of Fn14 in a linear pattern, indicating expression in the podocyte foot processes and increased Fn14 expression in the glomerular endothelial cells, seen as small circles (Fig. 3).

#### Histopathological classification score

Kidney biopsies were available for 39 patients. Histopathological Berden classification was performed on 28 biopsies. Twelve of the biopsies were classified as focal, eight as mixed, three as sclerotic and five as crescentic. There were no significant differences in sTWEAK or uTWEAK/Cr levels across these groups (Supplementary Table 1). All biopsy reports (*n* = 39) were subsequently reviewed for percentage of crescents. A significant correlation was found between the percentage of crescents and uTWEAK/Cr (*ρ* = 0.36, *P* = .03) (Fig. 2) but was not observed for sTWEAK.

## DISCUSSION

In this study, the role of TWEAK as a possible biomarker was investigated in a longitudinal cohort of AAV patients. We found that uTWEAK levels were higher in patients with active disease compared with at follow-up and correlated with disease activity assessed by BVAS. We also found an association between uTWEAK and albuminuria as well as the extent of crescentic changes on kidney biopsies. uTWEAK levels were elevated in patients with AAV kidney manifestations compared with those without. Our findings indicate a role for TWEAK in AAV and suggest that uTWEAK could be a potential non-invasive biomarker in AAV.

Our findings are in line with LN studies where higher levels of uTWEAK have been observed in active compared with inactive disease [30–32]. Interestingly, 14 AAV patients with kidney involvement were included in one of these studies as a disease control group and no difference was observed between uTWEAK levels in patients with AAV and LN [33], suggesting that uTWEAK may be a useful marker of kidney inflammation in general. uTWEAK levels have also been shown to be increased in other glomerular diseases [24]. TWEAK is a multifunctional cytokine, believed to promote tissue reparation and regeneration following acute injury, but it also induces tissue damage in the setting of chronic injury [34]. Fn14 and TWEAK have been shown to be upregulated in inflammation in kidney injury and appear to regulate inflammatory responses in tubular epithelial and mesangial cells [9, 35]. We observed increased staining of TWEAK in glomerular and tubular areas in kidney tissue from patients with crescentic pauci-immune necrotizing glomerulonephritis but not in control kidney tissue. This is similar to previously reported findings in patients with IgAN and AAV, where TWEAK and Fn14 were detected in glomerular crescents [24]. Elevated uTWEAK levels in patients with kidney involvement and the upregulated TWEAK expression in kidney tissue from patients with AAV as well as the correlation between uTWEAK levels and the extent of crescentic changes in our study indicate that the TWEAK/Fn14 pathway may be involved in the pathogenesis of ANCA-associated glomerulonephritis. A correlation was found between uTWEAK and the percentage of crescents on the kidney biopsies but we did not observe any association with histopathological Berden classes. These findings could indicate that histopathological parameters such as the extent of crescents are better indicators of the disease severity [36, 37].

We found that uTWEAK levels correlated with albuminuria, but not with other urine proteins (kappa, lambda and protein HC) at baseline, suggesting that uTWEAK levels do not reflect low molecular weight proteinuria. Most LN studies have failed to demonstrate a link between proteinuria and uTWEAK levels [33, 38, 39], although such a correlation has been reported in IgAN [24]. This discrepancy in findings may indicate that uTWEAK is not only a marker of the degree of proteinuria but reflects increased local inflammatory activity and regeneration in the kidney. Sasaki *et al*. on the other hand reported a correlation between uTWEAK levels and proteinuria in patients with glomerular diseases not characterized by infiltrative inflammation, such as minimal change disease and membranous nephropathy [24]. Whether elevated uTWEAK levels truly reflect local kidney injury and inflammation or rather a damaged glomerular filtration barrier remains therefore unclear.

In contrast to urinary findings, sTWEAK levels did not correlate with disease activity and no significant difference was found in levels between patients and controls. Similarly, a recently published cross-sectional study from our group showed no significant difference in sTWEAK levels when comparing patients with active and inactive AAV or AAV patients and controls [40]. However, we found a correlation between kidney function and sTWEAK. Serum TWEAK levels have been reported to be decreased in patients with kidney failure compared with healthy controls [41, 42]. A potential explanation for this correlation between sTWEAK and eGFR could be an increased uptake of TWEAK by its receptor, Fn14, which is upregulated in inflammatory disease states and injury [13, 14, 43]. Another hypothesis is an upregulation of CD163, which is the TWEAK scavenger receptor [44, 45]. A case study demonstrated that CD163 is abundantly expressed in acute kidney injury [46] and the expression of CD163 has been shown to be upregulated in kidney tissue in acute crescentic glomerulonephritis [47]. TWEAK binding to CD163 results in internalization of TWEAK, potentially decreasing sTWEAK concentrations [48, 49]. Interestingly, urinary CD163 has been suggested to be a potential biomarker in ANCA-associated glomerulonephritis [50]. Future studies are, however, needed to better delineate the relationship between CD163 and TWEAK in AAV.

We found no correlation between serum and urinary TWEAK levels, which further suggests that TWEAK may be upregulated specifically in the kidney during the acute phase of AAV. This is consistent with the results of Sasaki *et al*. on TWEAK levels in IgAN, where no correlation was found between serum and urinary TWEAK levels [24].

uTWEAK levels were higher in patients on immunosuppressive treatment at baseline and in patients who had received cyclophosphamide compared with those who had not. A positive correlation was also found between the cumulative GC dose and the uTWEAK levels at baseline. This may be due to the size of the cohort but could also be explained, at least partly, by higher disease activity in these patients. Supporting this we found a significant correlation between BVAS and the cumulative GC dose at inclusion.

The strength of our study is the relatively large and well-defined cohort of AAV patients with diverse organ manifestations and the longitudinal follow-up making it possible to observe changes over time and the effect of treatment. One of the limitations of this study is that the majority of the patients were already on induction treatment at inclusion. However as AAVs often are acute and potentially life- and organ-threatening conditions, patient recruitment in studies before treatment onset is challenging. However, the use of immunosuppressive treatment at inclusion did not seem to influence the uTWEAK/Cr levels at baseline. Another limitation is the lack of urine samples from population-based controls for comparison as well as the lack of a disease control group. Furthermore, the relatively small patient cohort with available kidney biopsies might affect the histopathological and clinical correlations.

In conclusion, this longitudinal study of AAV patients demonstrated that uTWEAK was increased at baseline compared with 6-month follow-up. uTWEAK levels also correlated with BVAS and albuminuria and were associated with kidney involvement and histopathological changes, whereas sTWEAK levels were not. These novel findings suggest that uTWEAK may be an interesting non-invasive biomarker to study further in longitudinal AAV follow-up and relapse studies.

## Supplementary Material

sfaf086_Supplemental_File

## Data Availability

The data underlying this article will be shared on reasonable request to the corresponding author.

## References

[1] Jennette JC, Falk RJ, Bacon PA et al. 2012 revised International Chapel Hill Consensus Conference Nomenclature of Vasculitides. Arthritis Rheum 2013;65:1–11. 10.1002/art.37715 23045170

[2] Westman KW, Bygren PG, Olsson H et al. Relapse rate, renal survival, and cancer morbidity in patients with Wegener's granulomatosis or microscopic polyangiitis with renal involvement. J Am Soc Nephrol 1998;9:842–52. 10.1681/ASN.V95842 9596082

[3] Aasarod K, Bostad L, Hammerstrom J et al. Renal histopathology and clinical course in 94 patients with Wegener's granulomatosis. Nephrol Dial Transplant 2001;16:953–60. 10.1093/ndt/16.5.953 11328900

[4] Hogan SL, Falk RJ, Chin H et al. Predictors of relapse and treatment resistance in antineutrophil cytoplasmic antibody-associated small-vessel vasculitis. Ann Intern Med 2005;143:621–31. 10.7326/0003-4819-143-9-200511010-00005 16263884

[5] Westman K, Flossmann O, Gregorini G. The long-term outcomes of systemic vasculitis. Nephrol Dial Transplant 2015;30:i60–6. 25601266 10.1093/ndt/gfu392

[6] Michaelson JS, Wisniacki N, Burkly LC et al. Role of TWEAK in lupus nephritis: a bench-to-bedside review. J Autoimmun 2012;39:130–42. 10.1016/j.jaut.2012.05.003 22727560 PMC3428508

[7] Kim SH, Kang YJ, Kim WJ et al. TWEAK can induce pro-inflammatory cytokines and matrix metalloproteinase-9 in macrophages. Circ J 2004;68:396–9. 10.1253/circj.68.396 15056843

[8] Maecker H, Varfolomeev E, Kischkel F et al. TWEAK attenuates the transition from innate to adaptive immunity. Cell 2005;123:931–44. 10.1016/j.cell.2005.09.022 16325585

[9] Justo P, Sanz AB, Sanchez-Nino MD et al. Cytokine cooperation in renal tubular cell injury: the role of TWEAK. Kidney Int 2006;70:1750–8. 10.1038/sj.ki.5001866 17003819

[10] Donohue PJ, Richards CM, Brown SA et al. TWEAK is an endothelial cell growth and chemotactic factor that also potentiates FGF-2 and VEGF-A mitogenic activity. Arterioscler Thromb Vasc Biol 2003;23:594–600. 10.1161/01.ATV.0000062883.93715.37 12615668

[11] Brown SA, Ghosh A, Winkles JA. Full-length, membrane-anchored TWEAK can function as a juxtacrine signaling molecule and activate the NF-kappaB pathway. J Biol Chem 2010;285:17432–41. 10.1074/jbc.M110.131979 20385556 PMC2878507

[12] Burkly LC, Michaelson JS, Zheng TS. TWEAK/Fn14 pathway: an immunological switch for shaping tissue responses. Immunol Rev 2011;244:99–114. 10.1111/j.1600-065X.2011.01054.x 22017434

[13] Sanz AB, Sanchez-Nino MD, Ortiz A. TWEAK, a multifunctional cytokine in kidney injury. Kidney Int 2011;80:708–18. 10.1038/ki.2011.180 21697814

[14] Burkly LC, Michaelson JS, Hahm K et al. TWEAKing tissue remodeling by a multifunctional cytokine: role of TWEAK/Fn14 pathway in health and disease. Cytokine 2007;40:1–16. 10.1016/j.cyto.2007.09.007 17981048

[15] Chicheportiche Y, Chicheportiche R, Sizing I et al. Proinflammatory activity of TWEAK on human dermal fibroblasts and synoviocytes: blocking and enhancing effects of anti-TWEAK monoclonal antibodies. Arthritis Res 2002;4:126–33. 10.1186/ar388 11879548 PMC83846

[16] Jakubowski A, Browning B, Lukashev M et al. Dual role for TWEAK in angiogenic regulation. J Cell Sci 2002;115:267–74. 10.1242/jcs.115.2.267 11839778

[17] Nakayama M, Ishidoh K, Kayagaki N et al. Multiple pathways of TWEAK-induced cell death. J Immunol 2002;168:734–43. 10.4049/jimmunol.168.2.734 11777967

[18] Nakayama M, Ishidoh K, Kojima Y et al. Fibroblast growth factor-inducible 14 mediates multiple pathways of TWEAK-induced cell death. J Immunol 2003;170:341–8. 10.4049/jimmunol.170.1.341 12496418

[19] Gomez IG, Roach AM, Nakagawa N et al. TWEAK-Fn14 signaling activates myofibroblasts to drive progression of fibrotic kidney disease. J Am Soc Nephrol 2016;27:3639–52. 10.1681/ASN.2015111227 27026366 PMC5118488

[20] Sanz AB, Izquierdo MC, Sanchez-Nino MD et al. TWEAK and the progression of renal disease: clinical translation. Nephrol Dial Transplant 2014;29:i54–62. 10.1093/ndt/gft342 24493870 PMC3968810

[21] Poveda J, Vazquez-Sanchez S, Sanz AB et al. TWEAK-Fn14 as a common pathway in the heart and the kidneys in cardiorenal syndrome. J Pathol 2021;254:5–19. 33512736 10.1002/path.5631

[22] Choe JY, Kim SK. Serum TWEAK as a biomarker for disease activity of systemic lupus erythematosus. Inflamm Res 2016;65:479–88. 10.1007/s00011-016-0930-5 26921306

[23] Wang C, Chen LL, Pan HF et al. Expression of human tumor necrosis factor-like weak inducer of apoptosis in patients with systemic lupus erythematosus. Clin Rheumatol 2012;31:335–9. 10.1007/s10067-011-1865-4 21968693

[24] Sasaki Y, Shimizu Y, Suzuki Y et al. TWEAK/Fn14 system and crescent formation in IgA nephropathy. BMC Nephrol 2015;16:27. 10.1186/s12882-015-0022-8 25885587 PMC4363378

[25] Schwartz N, Rubinstein T, Burkly LC et al. Urinary TWEAK as a biomarker of lupus nephritis: a multicenter cohort study. Arthritis Res Ther 2009;11:R143. 10.1186/ar2816 19785730 PMC2787265

[26] Stenvinkel P, Heimburger O, Paultre F et al. Strong association between malnutrition, inflammation, and atherosclerosis in chronic renal failure. Kidney Int 1999;55:1899–911. 10.1046/j.1523-1755.1999.00422.x 10231453

[27] Mukhtyar C, Lee R, Brown D et al. Modification and validation of the Birmingham Vasculitis Activity Score (version 3). Ann Rheum Dis 2009;68:1827–32. 10.1136/ard.2008.101279 19054820

[28] Levey AS, Stevens LA, Schmid CH et al. A new equation to estimate glomerular filtration rate. Ann Intern Med 2009;150:604–12. 10.7326/0003-4819-150-9-200905050-00006 19414839 PMC2763564

[29] Berden AE, Ferrario F, Hagen EC et al. Histopathologic classification of ANCA-associated glomerulonephritis. J Am Soc Nephrol 2010;21:1628–36. 10.1681/ASN.2010050477 20616173

[30] Xuejing Z, Jiazhen T, Jun L et al. Urinary TWEAK level as a marker of lupus nephritis activity in 46 cases. J Biomed Biotechnol 2012;2012:359647. 10.1155/2012/359647 22719208 PMC3375113

[31] Dong X, Zheng Z, Luo X et al. Combined utilization of untimed single urine of MCP-1 and TWEAK as a potential indicator for proteinuria in lupus nephritis: a case-control study. Medicine (Baltimore) 2018;97:e0343. 10.1097/MD.0000000000010343 29668584 PMC5916697

[32] Wang ZH, Dai ZW, Dong YY et al. Urinary tumor necrosis factor-like weak inducer of apoptosis as a biomarker for diagnosis and evaluating activity in lupus nephritis: a meta-analysis. J Clin Rheumatol 2021;27:272–7. 10.1097/RHU.0000000000001316 32028305

[33] Mirioglu S, Cinar S, Yazici H et al. Serum and urine TNF-like weak inducer of apoptosis, monocyte chemoattractant protein-1 and neutrophil gelatinase-associated lipocalin as biomarkers of disease activity in patients with systemic lupus erythematosus. Lupus 2020;29:379–88. 10.1177/0961203320904997 32041504

[34] Burkly LC. TWEAK/Fn14 axis: the current paradigm of tissue injury-inducible function in the midst of complexities. Semin Immunol 2014;26:229–36. 10.1016/j.smim.2014.02.006 24636536

[35] Hotta K, Sho M, Yamato I et al. Direct targeting of fibroblast growth factor-inducible 14 protein protects against renal ischemia reperfusion injury. Kidney Int 2011;79:179–88. 10.1038/ki.2010.379 20927042

[36] Brix SR, Noriega M, Tennstedt P et al. Development and validation of a renal risk score in ANCA-associated glomerulonephritis. Kidney Int 2018;94:1177–88. 10.1016/j.kint.2018.07.020 30385041

[37] Boud'hors C, Copin MC, Wacrenier S et al. Histopathological prognostic factors in ANCA-associated glomerulonephritis. Autoimmun Rev 2022;21:103139. 10.1016/j.autrev.2022.103139 35835443

[38] Moloi MW, Rusch JA, Omar F et al. Urinary MCP-1 and TWEAK as non-invasive markers of disease activity and treatment response in patients with lupus nephritis in South Africa. Int Urol Nephrol 2021;53:1865–73. 10.1007/s11255-020-02780-9 33459955

[39] Suttichet TB, Kittanamongkolchai W, Phromjeen C et al. Urine TWEAK level as a biomarker for early response to treatment in active lupus nephritis: a prospective multicentre study. Lupus Sci Med 2019;6:e000298. 10.1136/lupus-2018-000298 31168397 PMC6519400

[40] Manojlovic M, Juto A, Jonasdottir A et al. Microparticles expressing myeloperoxidase as potential biomarkers in anti-neutrophil cytoplasmic antibody (ANCA)-associated vasculitides (AAV). J Mol Med (Berl) 2020;98:1279–86. 10.1007/s00109-020-01955-2 32734361 PMC7447662

[41] Kralisch S, Ziegelmeier M, Bachmann A et al. Serum levels of the atherosclerosis biomarker sTWEAK are decreased in type 2 diabetes and end-stage renal disease. Atherosclerosis 2008;199:440–4. 10.1016/j.atherosclerosis.2007.10.022 18054361

[42] Carrero JJ, Ortiz A, Qureshi AR et al. Additive effects of soluble TWEAK and inflammation on mortality in hemodialysis patients. Clin J Am Soc Nephrol 2009;4:110–8. 10.2215/CJN.02790608 18945991 PMC2615702

[43] Burkly LC, Dohi T. The TWEAK/Fn14 pathway in tissue remodeling: for better or for worse. Adv Exp Med Biol 2011;691:305–22. 10.1007/978-1-4419-6612-4_32 21153335

[44] Yilmaz MI, Carrero JJ, Ortiz A et al. Soluble TWEAK plasma levels as a novel biomarker of endothelial function in patients with chronic kidney disease. Clin J Am Soc Nephrol 2009;4:1716–23. 10.2215/CJN.02760409 19820131 PMC2774962

[45] Ruiz-Ortega M, Ortiz A, Ramos AM. Tumor necrosis factor-like weak inducer of apoptosis (TWEAK) and kidney disease. Curr Opin Nephrol Hypertens 2014;23:93–100. 10.1097/01.mnh.0000437331.23794.81 24257157

[46] Martin Cleary C, Moreno JA, Fernandez B, Ortiz A et al. Glomerular haematuria, renal interstitial haemorrhage and acute kidney injury. Nephrol Dial Transplant 2010;25:4103–6. 20709744 10.1093/ndt/gfq493

[47] Li J, Liu CH, Xu DL et al. Significance of CD163-positive macrophages in proliferative glomerulonephritis. Am J Med Sci 2015;350:387–92. 10.1097/MAJ.0000000000000569 26379042

[48] Bover LC, Cardo-Vila M, Kuniyasu A et al. A previously unrecognized protein-protein interaction between TWEAK and CD163: potential biological implications. J Immunol 2007;178:8183–94. 10.4049/jimmunol.178.12.8183 17548657

[49] Moreno JA, Munoz-Garcia B, Martin-Ventura JL et al. The CD163-expressing macrophages recognize and internalize TWEAK: potential consequences in atherosclerosis. Atherosclerosis 2009;207:103–10. 10.1016/j.atherosclerosis.2009.04.033 19473660

[50] Moran SM, Scott J, Clarkson MR et al. The clinical application of urine soluble CD163 in ANCA-associated vasculitis. J Am Soc Nephrol 2021;32:2920–32. 10.1681/ASN.2021030382 34518279 PMC8806104

