# Supplementary material for: Urinary TWEAK reflects disease activity in ANCA-associated vasculitis
Source: Clin Kidney J. 2025 Apr 18;18(5):sfaf086. doi: 10.1093/ckj/sfaf086 (PMC12121554; doi:10.1093/ckj/sfaf086)
Supplement: sfaf086_Supplemental_File [file sfaf086_Supplemental_File.pdf]

Supplementary Table 1. Serum and urinary TWEAK in different histopathological classes (Berden classification)

	**Crescentic (n=5)**	**Focal (n=12)**	**Mixed (n=8)**	**Sclerotic (n=3)**	***P***
Serum TWEAK (pg/ml)	479,5 (308,8-981,7)	522 (455,2-728,3)	411,9 (273,5-803,3)	452 (191,8-612,8)	0,71
Urinary TWEAK/Cr* (ng/mmol)	8,1 (4,1-12,1)	4,2 (3,1-9,4)	8,6 (6,0-29,6)	4,9 (1,6-8,4)	0,21

Data presented as median (25th-75th percentile), Kruskal Wallis H-test.
TWEAK: TNF-like weak inducer of apoptosis
**Urine measurement available in 2 patients with crescentic class and 11 patients with focal class*
